# Epitope Characterization of an Aromatase Monoclonal Antibody Suitable for the Assessment of Intratumoral Aromatase Activity

**DOI:** 10.1371/journal.pone.0008050

**Published:** 2009-11-30

**Authors:** Yanyan Hong, Hongzhi Li, Jingjing Ye, Yasuhiro Miki, Yate-Ching Yuan, Hironobu Sasano, Dean B. Evans, Shiuan Chen

**Affiliations:** 1 Division of Tumor Cell Biology, Beckman Research Institute of the City of Hope, Duarte, California, United States of America; 2 Division of Information Sciences, Beckman Research Institute of the City of Hope, Duarte, California, United States of America; 3 Department of Pathology, Tohoku University Graduate School of Medicine, 2–1 Seiryou-machi, Aoba-ku, Sendai, Miyagi, Japan; 4 Oncology Research, Novartis Institutes for Biomedical Research, Novartis Pharma Ag, Switzerland; Baylor College of Medicine, United States of America

## Abstract

Immunohistochemistry is one of the most suitable methods for the detection of intratumoral aromatase in order to identify patients who may respond to aromatase inhibitor therapy in hormone-dependent breast cancer. Previous studies showed statistically significant correlation between results of immnuohistochemistry and biochemical analysis in carcinoma components stained by aromatase monoclonal antibody 677. In this study, determination of the antigenic peptides recognized by aromatase antibodies through epitope mapping, combined with the new knowledge on aromatase-reductase interaction, provide insights for understanding various immunostaining patterns using different aromatase antibodies. Our studies on aromatase-reductase interaction also provided critical information on how aromatase and reductase interact with each other on the endoplasmic reticulum membrane, and identified key residues, including K108 of aromatase, that are involved in the interaction with reductase. Through epitope mapping and taking into consideration the interference with aromatase immunohistochemical staining by NADPH-cytochrome P450 reductase, we demonstrated that monoclonal antibody 677 is a suitable antibody for an assessment of intratumoral aromatase activity in breast cancer patients for making clinical management decisions. These results also provide valuable information to identify new aromatase antibodies for immunohistochemical diagnosis of hormone-dependent breast cancer in future.

## Introduction

Aromatase is the rate-limiting enzyme in estrogen biosynthesis. Estrogen plays an important role in breast cancer development. Upon binding to estrogen, estrogen receptor activates transcription of its target genes, which are responsible for cancer cell proliferation in hormone-dependent breast tumors. Increased aromatase expression and activity have been reported in human breast tumor compared with normal breast tissue [1]–[3]. Intratumoral aromatase is a therapeutic target for the treatment of hormone-dependent breast cancer in post-menopausal women. Immunohistochemistry is one of the most suitable methods for the detection of intratumoral aromatase. Some studies have demonstrated the correlation between the response to aromatase inhibitor therapy and the amount of intratumoral aromatase activity or expression [4], [5]. Therefore, reliable aromatase antibodies for immunohistochemistry are of help in the characterization of hormone-dependent breast cancer in order to potentially identify post-menopausal patients with ER positive tumors who will respond to aromatase inhibitor therapy.

Several antibodies [1], [6]–[9] have been used to detect aromatase by immunohistochemistry but all of them are associated with the following limitations: (1) insufficient characterization of antibodies, (2) aromatase immnunoreactivity was evaluated by only one pathologist, (3) aromatase immunoreactivity in tissue sections were not scored or graded, (4) no correlations were examined between aromatase immunoreactivity and intratumoral aromatase activity [10]. Therefore, a multi-centre collaborative group has been established to generate and validate new aromatase monoclonal antibodies using purified recombinant GST-aromatase fusion protein as antigen for immunization of mice [11]. Their objective was to produce specific monoclonal antibodies (MCAs) against aromatase that are capable of detecting aromatase through immunohistochemistry of 10% formalin-fixed paraffin embedded sections of breast carcinomas and establishment of scoring systems which would be best correlated with biochemical assays of the same specimens. Twenty-three MCAs selected by biochemical assays were evaluated by immunohistochemistry of paraffin-embedded tissue sections including normal ovary and placenta, and a small series of 10 breast carcinomas. Further definitive characterization using 43 cases of breast cancer showed statistically significant correlation between results of immnuohistochemistry and biochemical analysis in carcinoma components stained by MCA 677, an antibody against native aromatase protein. Therefore, MCA 677 could be used in quantitative assessment of intratumoral aromatase activity in breast cancer patients for making clinical management decisions. To explain why MCA 677 is a better antibody, an epitope mapping is essential for a precise determination of which area of aromatase protein recognized by this antibody.

At present, aromatase antibodies have been engineered mainly against aromatase protein without the consideration of the interference of reductase *in vivo*. Aromatase belongs to the cytochrome P450 family, and forms an electron-transfer complex with its partner, NADPH-cytochrome P450 reductase, during the aromatization of androgen to estrogen. Reductase is composed of four domains: the FMN-binding domain, connecting domain, FAD-binding domain, and the NADP-binding domain, revealed by the crystal structure of reductase solved in 1997 [12]. During the aromatization reaction, electrons are transferred from NADPH, through FAD and FMN, to the heme of aromatase. The membrane binding site of reductase is situated around the V64 residue and near some hydrophobic patches of the surface, most likely, containing loops 250–281, 516–525, and 553–557 [12]. These membrane binding sites enable reductase to sit on the endoplasmic reticulum membrane, thus reductase adopts an orientation in which the FMN, FAD, and NADP binding sites are facing towards the cytoplasmic side. The crystal structure of human aromatase, solved recently by Ghosh and co-workers, represents a major breakthrough in aromatase research [13]. In their proposed membrane integration model, the opening to the active site access channel rests on the lipid bilayer of endoplasmic reticulum, allowing the steroidal substrate to enter into the active site directly from the bilayer. This model suggests that lipid integration/association sites include the N terminus up to the A helix and other loops near the C terminus [13]. A number of studies indicate the interactions between cytochrome P450 enzymes and reductase comprise interaction of the hydrophobic membrane binding portions and electrostatic attraction [14]–[17]. However, the molecular basis for the interaction between aromatase and reductase *in vivo* is not yet fully understood. In this study, determination of the antigenic peptides recognized by aromatase antibodies through epitope mapping, combined with the new knowledge on aromatase-reductase interaction, provide insights for understanding various immunostaining patterns using different aromatase antibodies.

## Results

### Immunohistochemical Analysis of Aromatase

**Figure 1:**
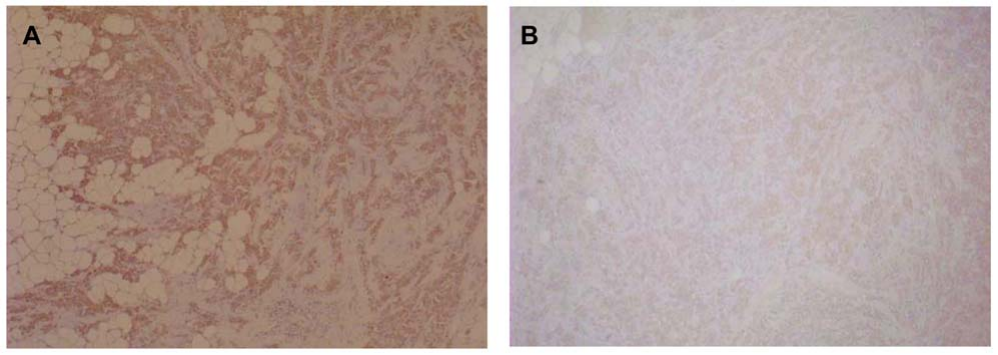
Immunohistochemical detection of aromatase in human breast carcinoma tissue specimens. (A) MCA 677; (B) F11.

Two MCAs 677 and F11 were used in this study. These two MCAs were generated and validated by a multi-centre collaborative group [10], [11] using recombinant baculovirus-expressed human aromatase protein as antigen; MCA 677 was raised against native protein and F11 against formalin-fixed protein. These two monoclonal antibodies could demonstrate aromatase immunoreactivity in breast cancer tissue specimens. Representative immunohistochemistry staining of human breast cancer specimens using these two MCAs is shown in Fig. 1. Furthermore, immunohistochemical staining results showed that a significant positive correlation was detected between aromatase immunohistochemistry stained with MCA 677 and aromatase biochemical activity in human breast carcinoma tissue specimens, while staining using MCA F11 as a primary antibody did not produce a positive correlation with aromatase activity (data not shown).

### Aromatase Epitope Analysis

To understand why MCA 677 is a better antibody than MCA F11 in the detection of aromatase in breast cancer tissues, we identified their peptide antigens through epitope mapping. One additional MCA, 2077, and one polyclonal antiserum were also included in this study. MCA 2077 was used as a reference control since it was raised using a peptide antigen–KALEDDVIDGYPVKKC, corresponding to amino acids 376–390 of human aromatase, plus an extra C-terminal cysteine residue [18]. The polyclonal antiserum was generated against functionally active human recombinant aromatase produced by the Chen laboratory [26].

**Figure 2:**
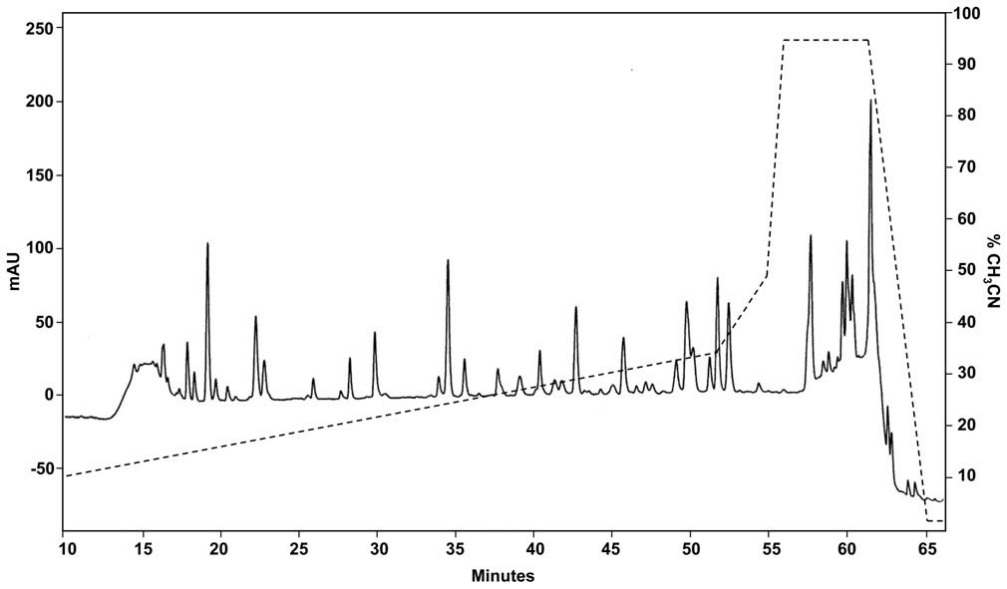
Reverse-phase HPLC tryptic peptide map of human recombinant aromatase. The dashed line represents CH_3_CN gradients.

**Figure 3:**
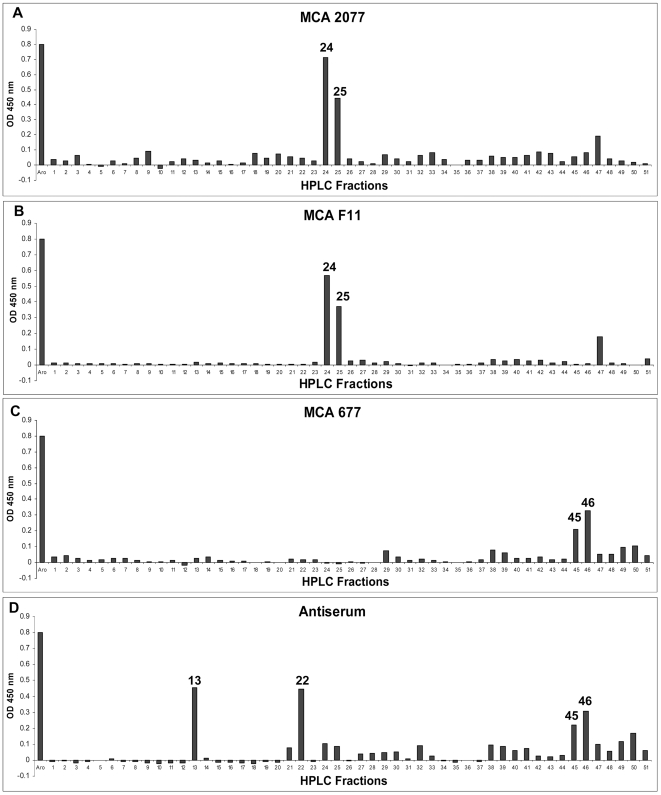
Peptide ELISA with four different aromatase antibodies.

**Figure 4:**
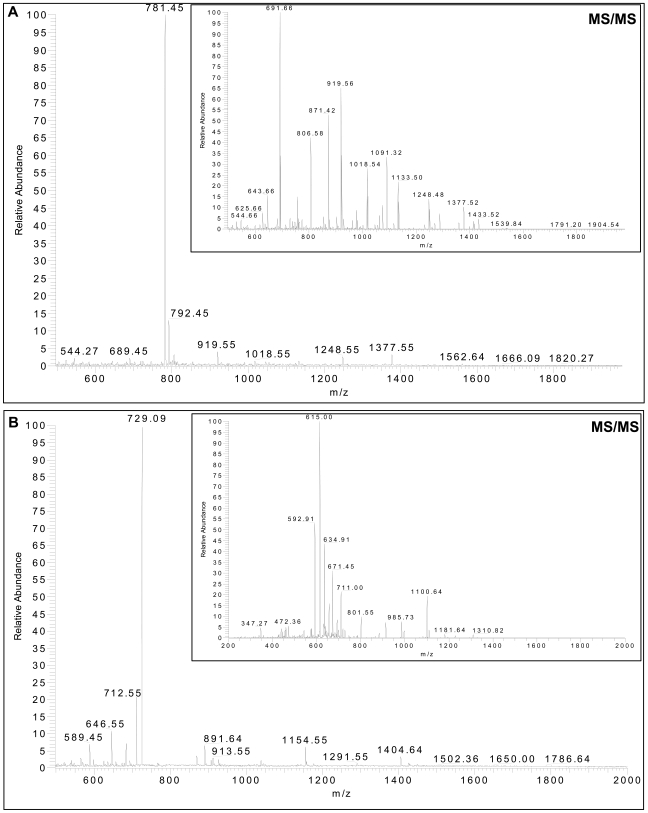
Peptide analysis using LTQ-FT mass spectrometer. (A) MS and MS/MS of HPLC fraction 25 that was recognized by MCAs 2077 and F11. The inset is the fragmentation (MS/MS) spectrum of the dominant peptide at m/z 781 [MH2 (2+)]. These experimental MS/MS fragment ions match to theoretical fragment ions produced by peptide ALEDDVIDGYPVKK, corresponding to amino acids 377–390 of human aromatase. (B) MS and MS/MS of HPLC fraction 46 that was recognized by MCA 677. The inset is the fragmentation (MS/MS) spectrum of the dominant peptide at m/z 729 [MH2 (2+)]. These experimental MS/MS fragment ions match to theoretical fragment ions produced by peptide DLKDAIEVLIAEK, corresponding to amino acids 250–262 of human aromatase.

Pure human recombinant aromatase protein was subjected to digestion by trypsin, and digested peptides were separated by reversed-phase HPLC using a PROTEO C18 HPLC column (Fig. 2). HPLC fractions were applied for ELISA using MCAs 677, F11, and 2077, and antiserum (Fig. 3). ELISA-positive fractions were analyzed by linear quadrupole ion trap Fourier transform mass spectrometer (LTQ-FT-MS) to identify the peptides in those fractions (Fig. 4). MCA 2077 recognized fractions 24 and 25. Fraction 24 contained the antigenic peptide–amino acids 376–390 of human aromatase, and fraction 25 contained the same peptide without an N-terminal lysine residue. MCA F11 recognized the same fractions as MCA 2077. MCA 677 recognized fractions 45 and 46, and these two fractions contained a same peptide–DLKDAIEVLIAEK, corresponding to amino acids 250–262 of human aromatase. The same peptide was also recognized by the antiserum generated in our laboratory. Besides this one, antiserum also recognized fraction 22, and it contained peptide–IHDLSLHPDETK, corresponding to amino acids 474–485 of human aromatase. We didn't find any peptides in faction 13, which was positive by ELISA assay using the antiserum. These epitopes are located on the surface of aromatase protein.

### Electrostatic Interaction between Aromatase and Reductase

To determine whether the interference of reductase contributes to different immunohistochemistry staining using MCAs #677 and F11, we investigate the interaction between aromatase and reductase. Previous studies suggest the interactions between reductase and cytochrome P450 enzyme comprise interaction of the hydrophobic membrane binding portions and electrostatic attraction [14]–[17]. Multiple sequence alignments of cytochrome P450s have shown that there are five homologous positions of positively charged amino acids which have been identified by protein modification or site-directed mutagenesis as being involved in the interaction with reductase [19]–[22]. In human aromatase, these candidate residues are K99, K108, K389/K390, K420, and R425 through sequence alignments with cytochrome P450 1A1, 1A2, 2B1, and 2B4. To check for potential electrostatic interaction between aromatase and reductase, we calculated the surface electrostatic potential of the crystal structures of aromatase (PDB: 3EQM) and reductase (PDB: 1AMO) by PYMOL. Surface electrostatic potential maps confirm that the FMN domain of reductase is mainly negatively charged and the surface of the heme proximal side of aromatase is highly positively charged. This agrees with the hypothesis that the FMN-binding domain of reductase provides a major surface for the electrostatic interaction with aromatase, since electrons exit from the FMN-binding domain and transfer to the heme of aromatase. This model also agrees with the assumption that the heme proximal side of aromatase faces reductase based on the fact that substrate binds to the heme distal side of aromatase.

### Computer Modeling Analysis of the Interaction between Aromatase and Reductase

**Figure 5:**
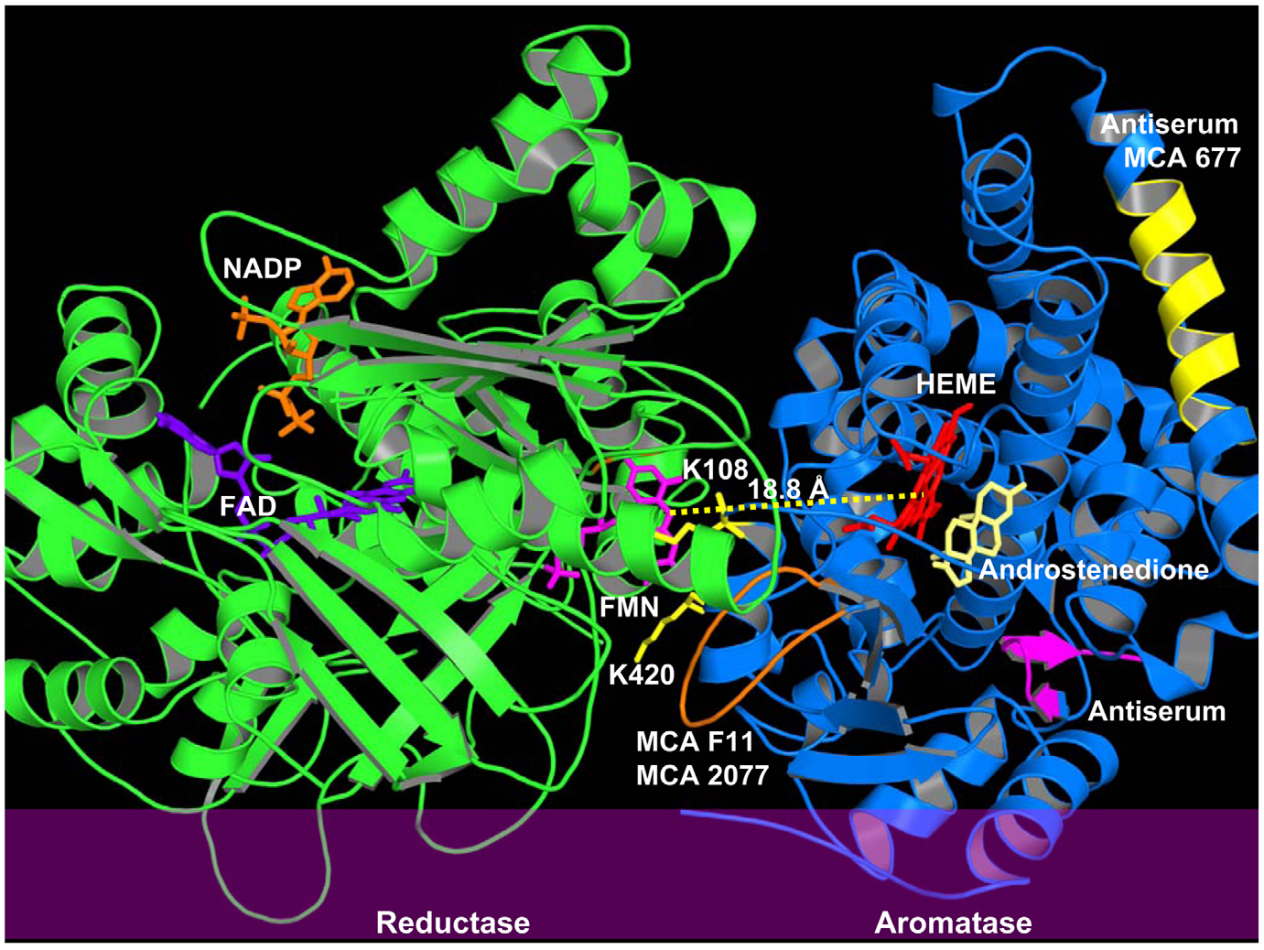
Computer-assisted docking model of the aromatase-reductase complex. A ribbon representation of the aromatase (*blue*)-reductase (*green*) complex showing its association with endoplasmic reticulum membrane (*purple*). The docking model was produced from ZDOCK [23] software version 3.0.1. NADP, FAD, FMN, HEME, and androstenedione are shown in *stick* representation, and colored in *orange*, *blue*, *purple*, *red*, and *yellow*, respectively. The distance between the N5 atom of FMN and the heme iron is 18.8 Å. Residues K108 and K420 of aromatase are colored in *yellow*. Epitopes are shown in *yellow* (MCA 677 and antiserum), *orange* (MCA 2077 and F11), and *purple* (antiserum).This figure was generated by PYMOL.

To better understand how aromatase interacts with reductase, we performed computer-assisted molecular docking. Direct rigid docking of the crystal structure of aromatase into the crystal structure of reductase using ZDOCK suggested that reductase may adopt a structural rearrangement when it forms the complex with aromatase. Flexible step-wise docking approach was then applied. Firstly, aromatase was docked with the FMN domain of reductase. The FAD and NADP domains were then docked into the complex. Protein-protein docking decoys were produced from ZDOCK [23] software version 3.0.1. A total of 13500 decoys were generated by selecting dense rotational sampling. The docking scores were then re-calculated based on the consensus of four equally-weighted measurements including: 1. distance between the FMN of reductase and the heme of aromatase; 2. whether the proximal surface of aromatase interacts with reductase; 3. whether aromatase forms ion pairs with reductase through predictive residues K99, K108, K389/K390, K420, or R425; 4. angle of two planes determined by the membrane binding surfaces of reductase and aromatase. The model with the highest docking score is shown in Fig. 5.

This docking model allows the N-terminal transmembrane domains and the membrane binding portions of two proteins to face the same orientation. The distance between the N5 atom of FMN and the heme iron is 18.8 Å, which is similar to the distance in the crystal structure of P450BM3, a self-sufficient enzyme with the heme domain and the reductase domain linked together on a single polypeptide [24]. It also allows electrostatic interactions between K108 of aromatase and N175/T177 of reductase and between K420 of aromatase and E115 of reductase. Residues N175, T177, and E115 of reductase are located within two important peptides, 175–182 and 109–130. Peptide 175–182 of reductase was predicted to be involved in the interaction between cytochrome c and reductase [12]. Carboxylate residues within the peptide 109–130 of reductase were identified as involved in the interaction with cytochrome P450 PB-b using radiolabelled nucleophile followed by proteolysis of the labeled protein [25].

### Validation of the Docking Model

**Figure 6:**
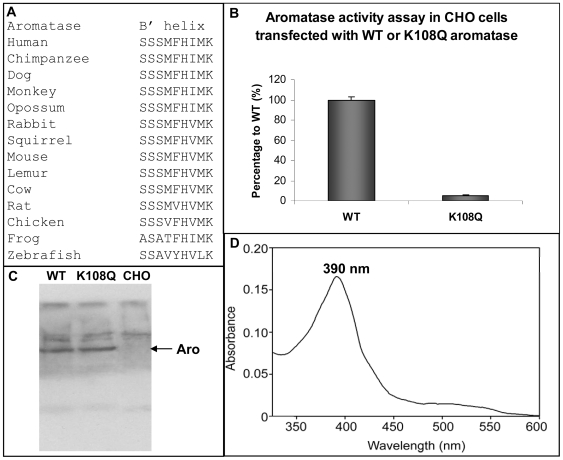
Validation of predicted function of residue lysine-108. (A) Amino acid sequences alignment of the B' helix of the aromatase family. (B) Aromatase activity assay of WT aromatase and the K108Q mutant in CHO cells. (C) Western blot analysis on whole-cell lysates from CHO cells control or transfected with WT or K108Q aromatase using aromatase polyclonal antibody. (D) Substrate binding spectrum of the K108Q aromatase in the presence of androstenedione.

Computer-assisted model predicts electrostatic interaction between residues N175/T177 of reductase and residue K108 of aromatase. Residue K108 is located at the edge of the B' helix (S^100^SSMFHIMK^108^) of aromatase. The B' helix is situated on the proximal surface of aromatase, and intrudes into the cleft between the FMN and FAD domains of reductase in the docking model, enabling K108 of aromatase to interact with N175/T177 of reductase. The amino acid sequence of the B' helix is well conserved among the aromatase family (Fig. 6A). To validate the predicted function of the K108 residue, we generated the K108Q mutant using site-directed mutagenesis. Aromatase activity assay using transfected CHO cells showed that aromatase activity of the K108Q mutant was significantly decreased (Fig. 6B). Western blot by polyclonal aromatase antibody confirmed that the mutant protein level is similar to the WT aromatase in transfected CHO cells (Fig. 6C).

**Figure 7:**
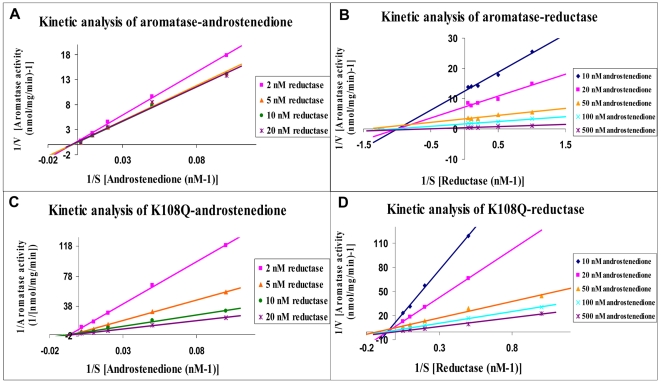
Lineweaver–Burk plots of aromatase kinetic analysis. (A) and (C). Kinetic analysis of recombinant aromatase or the K108Q mutant with substrate androstenedione (from 10 nM to 500 nM) in the presence of reductase (from 2 nM to 20 nM). (B) and (D). Kinetic analysis of recombinant aromatase or the K108Q mutant with substrate reductase (from 1 nM to 20 nM) in the presence of androstenedione (from 10 nM to 500 nM). Aromatase activity assay were performed by the tritiated water release method using [1β-^3^H(N)]-androstenedione with 50 nM pure aromatase protein.

In our laboratory, we have developed an experimental procedure to purify catalytically active recombinant aromatase [26]. We also express and purify full-length NADPH-cytochrome P450 reductase according to a procedure previously described [27]. To further determine whether the K108 residue is indeed involved in the interaction with reductase, we expressed and purified mutant protein from *E. coli* according to a procedure previously described [26]. The K108Q mutant showed a substrate binding spectrum in the presence of androstenedione, demonstrating that the K108Q mutation doesn't affect the substrate binding property of aromatase (Fig. 6D). Enzyme kinetic analysis using pure enzyme preparations were performed to determine the androgen substrate and reductase binding properties of the K108Q mutant. Aromatase activity in converting androstenedione to estrone or oxidizing reductase was measured by the release of tritiated water from [1β-^3^H(N)]-androstenedione. The *Km* (Michaelis-Menten constant) values of aromatase for androstenedione and reductase were estimated to be 200 nM and 1 nM from the Lineweaver–Burk plots (Figs. 7A & B). The *Km* values of the K108Q mutant for androstenedione and reductase were estimated to be 200 nM and 20 nM from Lineweaver–Burk plots (Figs. 7C & D). Although *Km* is not a dissociation constant that measures the binding strength, it can be used as an indicator of binding affinity. The K108Q mutation didn't change the binding affinity of the androgen substrate, however, it dramatically deceased the binding affinity of reductase. The aromatase-reductase interaction is highly sensitive to the modification of a surface basic residue K108, which supports a strong dependence on electrostatic interaction.

## Discussion

Both 677 and F11 reacted with aromatase by western blot, and gave specific cytoplasmic staining of epithelial cancer cells and minimal background staining [11]. However, a significant positive correlation between immunohistochemistry and aromatase biochemical activity was detected only in malignant epithelium stained with 677. Epitope mapping demonstrated MCA 677 recognized amino acids 250–262 of human aromatase, which are exposed in the cytoplasm, while MCA F11 recognized amino acids 376–390 of human aromatase, which are near the endoplasmic reticulum membrane and are covered by reductase from the docking model presented in this study (Fig. 5). It is likely that reductase interferes with the binding of MCA F11 to its epitope on aromatase in tissue specimens. On the contrary, the epitope for MCA 677 is away from the reductase binding site and the androgen access channel (predicted by Dr. Ghosh from the crystal structure of aromatase), thus MCA 677 is a good immunohistochemical antibody. However, MCA 677 would not be suitable for studies involving inhibition of the aromatase enzyme activity and this has been confirmed in our own studies (unpublished results). Epitope mapping combined with the knowledge on the binding orientation of reductase in the predictive aromatase-reductase complex provide insights into understanding why MCA 677 is a good antibody in the detection of aromatase in breast cancer tissues. These results also provide valuable information to identify new aromatase antibodies for immunohistochemistry in order to characterize hormone-dependent breast cancer in future.

Computer-assisted docking, combined with biochemical experiments including site-directed mutagenesis and kinetic analysis, led us to propose an aromatase-reductase complex model, and reveal that the FMN domain of reductase undergoes a structural rearrangement, allowing the proximal surface of aromatase to fit in the cleft between the FNN and FAD domains of reductase. Recently, crystal structures of a reductase variant with a four amino acid deletion in the hinge connecting the FMN and FAD domains reveal the FMN domain of reductase undergoes a structural rearrangement that separates it from the FAD domain and exposes the FMN domain, allowing it to interact with its redox partner, cytochrome P450 [28]. Hamdane and co-workers proposed that a similar movement occurs in the wild type enzyme in the course of transferring electrons from FMN to cytochrome P450, which has been hypothesized elsewhere [12], [24], [29]. We also identified key residues including K108 on the surface of aromatase that are involved in the interaction with reductase. The results from immunohistochemical staining, on the other hand, support our prediction for the aromatase-reductase binding model. Without a crystal structure of the aromatase-reductase complex, our studies provide critical information on how aromatase and reductase interact with each other on the endoplasmic reticulum membrane.

In conclusion, Aromatase inhibitor therapy is one of the hormonal treatments available to postmenopausal breast cancer patients. It has become important to identify patients who may respond to aromatase inhibitor therapy before it is initiated. The measurement of intratumoral aromatase content might provide parameters of response to aromatase inhibitors in addition to estrogen receptor measurement in surgical pathology specimens. In this study, determination of the antigenic peptides recognized by current aromatase antibodies through epitope mapping, and taking into consideration of the interference with aromatase immunohistochemical staining by reductase, we demonstrated that MCA 677 is a suitable antibody for an assessment of intratumoral aromatase activity in breast cancer patients for use in clinical management decisions.

## Materials and Methods

### Immunohistochemistry

Immunohistochemical analyses were principally performed employing the streptavidin-biotin amplification method, and have been previously described in detail [10], [11].

### Trypsin Digestion

Two milligram of pure human recombinant aromatase were concentrated into a thick, jelly-like mess using vacuum drying, then dissolved in 225 µl of 8 M deionized Urea solution plus 25 µl of 1 M NH_4_HCO_3_. The mixture was incubated at 56°C for 15 minutes after adding 25 µl of 45 mM DTT, followed by incubation at room temperature for another 15 minutes in the dark after adding 25 µl of 100 mM Iodoacetamide. Add 75 µl of 1 M NH_4_HCO_3_ to the solution, and dilute it with MilliQ-water to a final volume of 1 ml. Add 40 µl 1 µg/µl trypsin stock to give a 1∶50 trypsin:protein ratio by mass, and incubate overnight at 30°C. Quench the reaction with 50 µl of ultra-pure glacial acetic acid. Centrifuge the sample at 14,000 rpm for 10 minutes to remove the pellet, then store frozen until analyzed.

### Peptide Separation

Trypsin-digested aromatase peptides were separated by reversed-phase high-pressure liquid chromatography (HPLC) using a PROTEO C18 HPLC column (the Nest Group, Inc.). 50 µl of peptides mixture was loaded onto the column each time, and eluted with a step gradient of CH_3_CN (Buffer A = 0.1% TFA in H2O; Buffer B = 0.1% TFA in 10% H2O, 90% CH3CN; 2% B for 6 min, 2% B to 10% B for 3 min, 10% B to 35% B for 42 min, 35% B to 50% B for 4 min, 50% B to 95% B for 2 min, 95% B for 6 min, 95% B to 2% B for 3 min, and 2% B for 5 min). A total of 51 fractions were collected according to the absorbance at 214 nm.

### Enzyme-Linked Immunosorbent Assay (ELISA)

HPLC fractions were dried using vacuum drying, then dissolved in 205 µl 50 mM NaHCO_3_ buffer, pH 9.0. Fifty micromole of the each sample were added into a 96-well ELISA plate. After the incubation at 4°C overnight, remove antigen, add 400 µl blocking buffer (PBS +1% BSA +0.02% azide), and incubate the plate at room temperature for 2 hours. Remove the blocking buffer, wash once with 200 µl PBS plus 0.02% azide, add 50 µl antibody diluted in blocking buffer (MCA 677 = 1∶200, MCA F11 = 1∶200, MCA 2077 = 1∶200, antiserum = 1∶500), and incubate the plate at room temperature for 1.5 hours. Remove the antibody solution, wash three times with 200 µl PBS plus 0.05% Tween-20, add 50 µl anti-mouse or anti-rabbit HRP conjugated secondary antibody with 1∶1000 dilution in blocking buffer, and incubate the plate at room temperature for 1.5 hours. Remove the secondary antibody solution, wash three times with 200 µl PBS plus 0.05% Tween-20, add 50 µl DY999 substrate (R&D systems, Inc.), incubate the plate at room temperature for 20 min, add 50 µl 5.7% H_2_SO_4_, mix, then read OD at 450 nm using SpectraMax M5 plate reader (Molecular Devices).

### UV-Vis Spectral Analysis

Absorption spectrum of aromatase-androstenedione was measured by a UV-1700 PharmaSpec UV-Vis Spectrophotometer (Shimadzu Scientific Instruments, Columbia, MD) with a 1-cm quartz cuvette. Buffer containing 50 mM potassium phosphate (pH 7.4) and 20% glycerol was used as spectral reference. Sample contained 0.1 mg/ml pure K108Q mutant protein with 2 µM androstenedione in reference buffer.

### Kinetic Analysis of Aromatase

Aromatase activity was measured by the release of tritiated water from [1β-^3^H(N)]-androstenedione. *In vitro* aromatase activity assay was reconstituted with 50 nM pure human recombinant aromatase in a 200-µL reaction solution containing 67 nM potassium phosphate (pH 7.4), 0.1% BSA, 10 µM progesterone, pure rat reductase, and [1β-^3^H(N)]-androstenedione at 37°C for 20 min. The concentration of pure rat reductase ranged from 1 nM to 20 nM. The concentration of [1β-^3^H(N)]-androstenedione was ranged from 10 nM to 500 nM. The incubation was initiated by the addition of 300 µM NADPH, and terminated by the addition of 50 µl 20% trichloroacetic acid. The reaction solution was mixed with charcoal-dextran to remove any trace amount of unreacted substrate. After centrifugation, the radioactivity of the supernatant was counted by a liquid scintillation counter (LS 6500; Beckman Coulter, Inc., Fullerton, CA). The *Km* and *Vmax* values of aromatase were determined from Lineweaver–Burk plots. Each point represents the mean of triplicate experiments.

### Site-Directed Mutagenesis

Aromatase mutants were generated by the QuikChange site-directed mutagenesis kit (Stratagene, La Jolla, CA) using WT aromatase expression plasmids pHβ-Aro [30] or pET3b-Aro [26] as template. All mutations were verified by DNA sequencing. For stable cell transfection, mutant pHβ-Aro plasmid was transfected into Chinese Hamster Ovary (CHO) cells using lipofectamine 2000 reagent (Invitrogen, Carlsbad, CA). After two weeks of G418 selection, transfected cells were maintained in media containing 1 mg/ml G418. For protein expression, mutant pET3b-Aro plasmid was transformed into BL21 (DE3) *E. coli* strain.

### In-Cell Aromatase Activity Assay

In the in-cell aromatase assay, aromatase-transfected CHO cells were seeded in six-well plates, and 1 mL serum-free media containing 100 nM [1β-^3^H(N)]-androstenedione was added to each well. After one hour of incubation at 37°C, the media were mixed with charcoal-dextran to remove any trace amount of unreacted substrate. After centrifugation, the radioactivity of the supernatant was counted by a liquid scintillation counter. To determine protein concentration, cells remaining in each well were solubilized with 0.5 M NaOH and subjected to the Brad-ford assay method.

### Western Blotting

Aromatase-transfected CHO cells were cultured in 60-mm dishes and lysed in 300-µL SDS lysis buffer containing 62.5 mmol/L Tris-HCl (pH 6.8), 2% w/v SDS, 10% glycerol, 50 mmol/L DTT, and 0.01% bromophenol blue. Sixty micrograms of lysate were resolved on a 10% SDS-PAGE gel and then transferred onto a polyvinylidene difluoride membrane (Millipore). The membrane was blocked in a blocking buffer (5% w/v nonfat dry milk in TBST) for 1 hour at room temperature, incubated with aromatase antiserum at 1∶500 dilution at 4°C overnight, then incubated with mouse anti-rabbit horseradish peroxidase conjugated secondary antibody at 1∶5000 dilution (Santa Cruz Biotechnology) for 1 hour.
